# Analysis of Patient Cues in Asynchronous Health Interactions: Pilot Study Combining Empathy Appraisal and Systemic Functional Linguistics

**DOI:** 10.2196/40058

**Published:** 2022-12-20

**Authors:** Elena Rey Velasco, Hanne Sæderup Pedersen, Timothy Skinner, Impact Diabetes B2B Collaboration Group

**Affiliations:** 1 Department of Psychology University of Copenhagen Copenhagen Denmark; 2 Liva Healthcare Copenhagen Denmark; 3 Department of Nordic Studies and Linguistics University of Copenhagen Denmark; 4 See Acknowledgements Dublin Ireland

**Keywords:** telehealth, telecoaching, asynchronous communication, empathy, systemic functional linguistics, communication, health promotion, coding, linguistic analysis, user experience, coach-user interaction, tool development, lifestyle-related disease

## Abstract

**Background:**

Lifestyle-related diseases are among the leading causes of death and disability. Their rapid increase worldwide has called for low-cost, scalable solutions to promote health behavior changes. Digital health coaching has proved to be effective in delivering affordable, scalable programs to support lifestyle change. This approach increasingly relies on asynchronous text-based interventions to motivate and support behavior change. Although we know that empathy is a core element for a successful coach-user relationship and positive patient outcomes, we lack research on how this is realized in text-based interactions. Systemic functional linguistics (SFL) is a linguistic theory that may support the identification of empathy opportunities (EOs) in text-based interactions, as well as the reasoning behind patients' linguistic choices in their formulation.

**Objective:**

This study aims to determine whether empathy and SFL approaches correspond and complement each other satisfactorily to study text-based communication in a health coaching context. We sought to explore whether combining empathic assessment with SFL categories can provide a means to understand client-coach interactions in asynchronous text-based coaching interactions.

**Methods:**

We retrieved 148 text messages sent by 29 women who participated in a randomized trial of telecoaching for the prevention of gestational diabetes mellitus (GDM) and postnatal weight loss. We conducted a pilot study to identify users' explicit and implicit EOs and further investigated these statements using the SFL approach, focusing on the analysis of transitivity and thematic analysis.

**Results:**

We identified 164 EOs present in 42.37% (3478/8209) of the word count in the corpus. These were mainly negative (n=90, 54.88%) and implicit (n=55, 60.00%). We distinguished opening, content and closing messages structures. Most of the wording was found in the content (n=7077, 86.21%) with a declarative structure (n=7084, 86.30%). Processes represented 22.4% (n=1839) of the corpus, with half being material (n=876, 10.67%) and mostly related to food and diet (n=196, 54.92%), physical activity (n=96, 26.89%), and lifestyle goals (n=40, 11.20%).

**Conclusions:**

Our findings show that empathy and SFL approaches are compatible. The results from our transitivity analysis reveal novel insights into the meanings of the users’ EOs, such as their seek for help or praise, often missed by health care professionals (HCPs), and on the coach-user relationship. The absence of explicit EOs and direct questions could be attributed to low trust on or information about the coach’s abilities. In the future, we will conduct further research to explore additional linguistic features and code coach messages.

**Trial Registration:**

Australian New Zealand Clinical Trials Registry (ANZCTR) ACTRN12620001240932; https://www.anzctr.org.au/Trial/Registration/TrialReview.aspx?id=380020

## Introduction

Noncommunicable diseases (NCDs) account for 73.6% of deaths worldwide. These lifestyle-related diseases, such as cardiovascular diseases (CVDs), some cancers, respiratory diseases, and diabetes, are among the most common causes of death and disability [1]. The rapid increase in NCD rates is a global disease burden in both developed and developing nations. However, we now know that these NCDs can be prevented, or substantially delayed, by changes in lifestyle (eg, factors such as diet, physical activity, stress, and sleep), as shown in numerous trials [2-4]. Research has demonstrated that with appropriate individual and group support, individuals can achieve significant weight loss and sustainable changes in lifestyle [5,6]. Nonetheless, the high incidence of NCDs and the limited resources to deliver best-practice behavior change programs make large-scale prevention programs challenging. The need for cost-effective alternatives has led some countries, such as the United States [7] and the United Kingdom [8], to seek new strategies to promote health behavior changes.

To address the challenges of scalability and cost-effectiveness, NCD prevention programs are increasingly using digital technologies. One technology that facilitates access to prevention programs is telehealth, which is the use of video or audio technologies to deliver a health intervention. Telehealth has the potential to reduce health care costs and increase the scope of these programs, as it can substitute or supplement in-person visits when personal attendance is not possible (eg, patients living in rural areas [9,10]). Using telehealth to deliver face-to-face behavior change programs has been shown to be as effective as in-person programs for NCDs [11,12]. For these reasons, the field of telehealth has experienced substantial growth over recent years, and the COVID-19 pandemic has accelerated the process, with programs for mental health, rehabilitation, and medical consultations showing rapid increases in usage [13-15].

In the context of disease prevention programs, this approach is increasingly referred to as telehealth coaching in order to distinguish it from the delivery of more traditional telehealth services. Telehealth coaching uses an integrative health coaching (IHC) approach. IHC connects the coaching intervention with the individual’s personal values and sense of purpose [16]. Instead of being instructed on how to reach their goals, the coach provides the user, or person being coached, with the knowledge, skills, and confidence to perform autonomously [17]. These telehealth coaching programs combine multimodalities of digital technology to support people in achieving their lifestyle goals in a synchronous or asynchronous form. Traditional synchronous interactions use real-time, face-to-face meetings, telephone calls, or video calls [18], and asynchronous interactions consist of the exchange of texts, audio, or video messages that the user can access and review later [19]. At the same time, health coaching allows for only human [14,20], only automated [21,22], or hybrid [23-25] modalities. Although all of these have shown positive results, it is still not clear which one is more effective [26]. A recent meta-analysis showed that automated digital interventions (ADIs) are a good addition to weight loss coaching interventions and results are more effective when the coaching program duration is shorter [27].

Generally, digital health coaching interventions follow a prespecified framework, such as manuals or guidelines, based on the current evidence on behavior change [28] and psychosocial theories [29,30]. Evaluating whether coaches are delivering a program as intended is key to ensuring a homogenous and effective intervention, and telehealth coaching poses unique challenges in this regard due to its multiple modalities [31]. Although there is increasing research exploring the fidelity of such programs, current research work has focused on synchronous, face-to-face interventions delivered by coaches [32-34]. This research typically quantifies the behavior change techniques (BCTs) delivered by the coach and to some extent the way in which these interventions are realized. State-of-the-art findings show a predominant focus on the coaches’ performance and users’ outcomes without accounting for the users’ cues and responses [35], in addition to inconsistencies in fidelity reporting [36]. With increasing drivers for efficiency and the use of responsive artificial intelligence (AI) systems, the use of asynchronous interactions to support health coaching is growing. However, there is little research on these asynchronous interactions and a clear need to understand their nature and how to optimize them. Although asynchronous interventions are delivered through audio or video messages on a digital platform, the most common form of interaction is through the exchange of text messages. A coaching platform can be automatized to send scheduled messages (eg, reminders). There is a body of research on the use of automated messages to remind, prompt, or nudge healthier behavior, which demonstrates their potential [37], effectiveness [38], and language used [39,40] in text-based behavior change interventions. However, these messages represent a 1-way communication from the coaching platform to the individual. The users participating in these telehealth coaching programs also communicate directly to their coach or AI coaching platform. Their text messages can be responded to by an AI-based system (eg, chatbots) or by their coach (ie, individually crafted correspondence).

Nonetheless, there is a wealth of literature on the effectiveness of traditional synchronous, face-to-face patient-provider interactions where researchers share an overall concern for the quality of asynchronous consultations [41], as well as for the quality of the relationship developed [42]. In this context, the concepts of empathy, sympathy, and compassion in health care are crucial in the patient-provider relationship but sometimes confused with one another [43]. Compassion is a deep awareness of another person’s suffering, along with the wish to relieve it [44], whereas empathy is the cognitive skill to understand and “feel with” the patient. Some authors have identified the dimensions of cognitive, affective (relegated to sympathy), and emotional within the definition of empathy [45]. Other authors, such as Piasecki, see clinical empathy as “the ability to understand and participate in another person’s feelings and emotional state, while sympathy describes the listener’s feelings without understanding or sharing the patient’s emotions” [46]. The positive effect of clinical empathy on patient outcomes has been documented across psychological, sociological, therapeutical, and behavioral disciplines [47,48] and should be preserved in text-based, asynchronous interventions. An empathetic response is important for building a therapeutic alliance in psychotherapy, and effective relational skills are essential in behavior change programs for promoting health outcomes. Thereby, an empathic frame is a good start point when coding asynchronous messages. There are a number of tools in patient-provider communication for identifying opportunities for empathic responses [49], showing how providers often miss these opportunities [50], and providing advice to prevent it [51]. According to a review by Epstein et al [52], patient-centered communication (PCC) comprises “(1) eliciting and understanding the patient's perspective—concerns, ideas, expectations, needs, feelings and functioning, (2) Understanding the patient within his or her unique psychosocial context, (3) Reaching a shared understanding of the problem and its treatment with the patient that is concordant with the patient's values, and (4) Helping patients to share power and responsibility by involving them in choices to the degree that they wish.” Epstein’s arguments are present when expressing empathy in a health care context. However, this approach has not been informed by our understandings of language and, in particular, the functions of language.

Pounds [53] presented an empathy appraisal approach, supported by previous discourse analysis studies based on systemic functional linguistics (SFL), to explore the expressions of empathy in PCC. Nonetheless, it is surprising that her approach does not incorporate SFL into this patient-provider communication analysis. According to SFL theory, developed by Halliday and Matthiessen [54], language in itself has a communicative and a meaning potential that is realized through language production, and that language in itself is social semiotics, an approach to communication that aims to comprehend how individuals in particular social contexts interact through a variety of means. The goal of studying communication from this angle is to classify the semiotic decisions that communicators are able to make [55]. The empathy opportunities (EOs) that Pounds considered in her empathy appraisal may be further informed by these choices. Suchman [56] defined implicit EOs as “patient statements from which a clinician might infer an underlying emotion that has not been explicitly expressed” and explicit EOs as “statements about situations or concerns that might plausibly be associated with an emotion.” To fully grasp the meaning potential of the patients’ linguistic choices whenever they express an EO, however, we must first understand the 3 SFL metafunctions that construe that meaning: the ideational metafunction, which describes the speaker’s inner and outer experience; the interpersonal metafunction, which concerns the relationship between the speaker and the recipient as well as between the speaker and their message; and the textual metafunction, which is used to interpret the text as a text and not just as a cluster of words or clauses [57]. The transitivity system is a component of the ideational metafunction and goes further than the distinction between transitive and intransitive verbs. A transitivity analysis explores how the speaker construes their experience of the world. The processes, participants implicated, and circumstances of this experience are all part of the transitivity system. Processes are realized by the verbal group of the clause and can be classified as material, mental, relational, verbal, existential, or behavioral [58]. We provide a further description of the process categories in the Methods section. Several researchers have chosen this approach for a quantitative analysis of written discourse in literature, news, and social media texts [59-61]. Matthiessen’s [62] work adds valuable insights into the use of SFL in health care contexts and PCC. Pounds and De Pablos-Ortega [63] foresee the combination of the empathy appraisal approach and SFL categories to better understand patients’ (or users’) perspectives and to improve doctors’ (or experts’) communicative strategies in online counseling. Additionally, the experiential metafunction, which is embedded in the ideational metafunction, describes how the speaker uses language to communicate their perception of themselves and the world. For example, Fosgerau et al [64] examined the choices of patients with depression in the transitivity system. Furthermore, this system is the most basic SFL grouping used to quantify the experiential meaning expressed in a text message–based interaction systematically. Thus, a combination of the empathic qualities’ identification in a message and its functional grammar analysis may provide a start point for coding asynchronous messages. Results from this approach would subsequently lead to the identification of an appropriate coaching response.

Thereby, in this paper, we seek to explore whether Pounds’ empathy appraisal and SFL approaches have utility in coding asynchronous text messages in a health context. To that end, we conduct a pilot study to analyze a data set of messages posted by users of a telehealth coaching program. We then discuss how the findings may be used to inform optimal coaching responses to those messages.

## Methods

### Study Design

We coded a sample of 148 messages sent by 29 women from March 7 to June 21, 2021, on a telehealth coaching platform. The study population was an Irish cluster that belongs to an ongoing randomized trial on a telehealth coaching intervention for the prevention of gestational diabetes mellitus (GDM) and postnatal weight loss in 800 women in Australia, Ireland, the United Kingdom, and Spain (Bump2Baby and Me, protocol registration no. ACTRN12620001240932) [65]. The analyzed messages were the first 148 messages sent by the first 29 participants enrolled in the study, who had thus been in the intervention for a period between 0 and 15 weeks. Participants (users) were onboarded after a synchronous initial consultation with their health coach, and then, they received an average of 15 automated messages between enrolment and birth, which included educational material on lifestyle, well-being, and nutrition. Users also received nonautomated messages from their coach, which accounted for an average of 4 weekly, 4 biweekly, and 3 monthly tailored messages before birth. Coach messages included comments on the users’ progress and lifestyle goals, as well as providing educational content and counseling. These communications were based on a predefined structure and a framework grounded on the BCT taxonomy [28] and the motivational interviewing approach [66]. We imported these 148 user-sent messages to NVivo 12 Plus (QSR International), a qualitative analysis software program [67], and then coded them according to the empathy appraisal [53] and SFL [54] categories explained later. Author ERV performed 2 rounds of the coding process for all categories and discussed the issues with a second coder (author HSP).

### Ethical Considerations

The Bump2Baby and Me trial, where the authors are authorized researchers, is the source of the data set that was examined. Ethical approval was obtained, and all study participants provided written informed consent for the use of their data for research purposes, provided the findings were presented anonymously. Ethical approval was granted for all study sites (Dublin: National Maternity Hospital Ethics Committee ref EC18.2020; Bristol: Wales Research Ethics Committee ref 21/WA/0022; Granada: CEIM/CEI Provincial de Granada; Melbourne: Monash Health Human Research Ethics Committee ref RES-20-0000-892A). The data used in the study belongs only to the Irish arm of the study (Dublin). More information concerning these ethical considerations can be found in the published study protocol [65].

### Empathy Appraisal Categories

We assessed empathy according to the “appraisal” dimensions of empathy in doctor-patient interactions described by Pounds [53], where patients’ expression of feelings and views are categorized as the following EOs:

- Explicit expressions of negative feelings, such as an emotive behavior or a mental state (“I cried when I found out”).
- Implicit expression of negative feelings through reference to a negative experience, such as fear, confusion, anxiety, or sadness (“It’s been 3 days and I haven’t heard back from my GP”).
- Explicit expression of negative judgment (others or self; “She is such an irresponsible person”).
- Implicit expression of negative judgment (others or self; “I could have done better”).
- Explicit or implicit expression of positive self-judgment (“I am eating healthier than ever!”).
- Explicit expression of negative appreciation (things, events, actions; “The dinner was so boring”).
- Implicit expression of negative appreciation (things, events, actions; “I am not sure this is something for me”).

### Message Structure

We used a message structure to explore how each message was organized and whether it affected participants’ expressions of empathy. The main categories were *opening, content, and closing*, according to previous research on written messaging dynamics [68,69] to illustrate the text structure or “reading path” [70,71]. During the analysis, we created 2 more categories: *full structure* (Textbox 1), for messages using all 3 categories, and *single structure* (Textbox 2), for messages using only 1 of them.

**Full-structure examples.**

**Example 1**

Opening: “Hi (coach name), hope you're well. Quick question for you.

Content: I weigh myself every Monday morning for the study and I've actually lost weight over the last few weeks. Just 0.15kg. Should I be worried as I read from 15 weeks I should be putting on a pound a week!

Closing: Thanks a mill!”

**Example 2**

Opening: “Hi (coach name) hope your week is going well (emoji)

Content: so far mine is. Nausea has eased big time in the past 10 which is great and I’ve been having my evening meal. Still need to work on time out for a book etc (emoji) a work in progress. It would be great if you could send me some stretching to over the next few weeks to try keep the body somewhat limber. Find my hips can be a bit creeky or sore in the morning so maybe something to assist?

Closing: Thank you (participant name)”

**Single-structure examples.**

**Examples**

- “I think I would like to re configure my goals regarding exercise. If I could measure my steps that would probably be a good start to keep tabs on myself? What do you think?”
- “Thank you very much for all the information (coach name)”
- “Pilates starts this eve with elbowroom (emoji)” (attached image)

### Sentence Structure

We based the sentence structure categories on Halliday’s [54] systemic functional grammar according to speech function. In a declarative sentence, the subject comes before the finite (verb). In an interrogative sentence, the finite comes before the subject. Lastly, the subject is implicit in an imperative sentence [54]. Throughout the analysis, we found that some sentences had the speech function of a question realized by a declarative structure. Halliday [54] previously described this phenomenon regarding the relationship between the sentence structure and the 4 speech functions *offer*, *command*, *statement*, and *question*. As a result, we created a fourth category to account for it (Table 1).

**Table 1:** Sentence structure categories and examples.

Sentence structure category	Example
Declarative	“I signed up for a 4 week yoga class”
Interrogative	“What do you think?”
Imperative	“Please send them to me”
Declarative structure, question function	“I would like to check with you whether you have got any video of pelvic floor exercises”

### Processes

We used Halliday’s [54] classification to define the process categories. A process is realized by the verb and contributes to the speaker’s construal of experience. We coded each clause according to the *material, mental, relational, behavioral, verbal,* and *existential* process categories (Table 2): *Material* processes construe the actions of doing and happening. *Mental* processes account for sensing. *Relational* processes are used to characterize and identify. *Behavioral* processes represent outer manifestations of human inner workings, such as consciousness and physiological processes. *Verbal* processes refer to the language form and use, such as saying and meaning. Lastly, *existential* processes represent existence or happening [54].

**Table 2:** Process categories and examples.

Process category	Example
Material	“We made a pumpkin cake”
Mental	“I have just read your book” “She is considering your offer”
Relational	“The weather was very nice” “I have a blue coat”
Behavioral	“I will have a look”
Verbal	“We talked about the meeting”
Existential	“There is a shop around the corner”

### Transitivity Analysis

Halliday’s [72] concept of transitivity supplements the differentiation between transitive and nontransitive verbs. This differentiation depends on the presence or absence of an object that completes the process meaning [72]. Through the choices in the transitivity system, the speaker construes their experiences of the external world and the internal world of their consciousness. This system considers the participants involved, as well as the surrounding circumstances [73]. Thereby, transitivity allowed us to explore the construals of experience in the corpus by identifying the processes and participants [74].

## Results

### Participants’ Demographics and Program Details

Table 3 shows a description of the users’ demographics and program details. The mean age was 37.59 years (SD 3.69), and the BMI was overall normal (mean 25.82, SD 5.68). Regarding the telehealth coaching program details, users had been on the program for a mean of 80.76 days (SD 30.47) and had sent a mean of 2.27 messages (SD 1.19) at the time of our analysis. In contrast, coaches had sent a mean of 7.62 messages (SD 1.82). The most common goals set by the users were related to physical activity (n=27, 93.1%), diet (n=24, 82.8%), and the number of steps (n=21, 72.4%). Because this was a coaching program for pregnant women, weight was not a frequent goal (n=7, 24.1%) and coaches were encouraged not to promote it. Users could manually add any lifestyle-related goal into the life goals category (n=15, 51.7%) on the platform, such as “meditate in the morning,” “go to bed before midnight,” or “read a book for 20mins.”

We present an overview of the coding results in Table 4 as the number of coded references (occurrences), word count, and word count percentages. We identified 164 EOs, accounting for 42.37% (3478/8209) of the corpus. Negative empathic statements were the most present (n=2026, 24.68%), mostly through an implicit approach (n=1442, 17.57%) as an *implicit negative appreciation of things, events, or actions* (n=987, 12.02%). This implicit approach rate was similar to that of the *explicit or implicit expression of positive self-judgment* category (n=1481, 18.04%). We did not identify any explicit expression of negative judgment about others or self.

*Content* was the predominant structural component (n=7077, 86.21%). Nearly half of the messages (n=4011, 48.86%) included all 3 structural components (*opening*, *content*, and *closing*), while 5.26% (n=432) were identified as a *single message*, including 1 of the components (*opening*, *content*, or *closing*). In some cases, a user sent more than 1 message at the same time, resulting in the structural components being divided. We conducted a separate analysis comparing full- and single-structured messages that showed no differences for EOs, sentence structure, and processes.

Sentence structure coding revealed that the preferred sentence structure was *declarative* in this corpus (n=7084, 86.30%). This indicated that participants used these message exchanges to narrate, describe, or state rather than to request for information, guidance, or support. However, 9.43% (774/8209) of the corpus was not coded, because it did not meet the definition of a clause as previously explained (greetings, thanking, laughter, emojis, links in between independent sentences, vocatives), and was labeled as “other.”

Further, *processes* accounted for 22.40% (n=1839) of the corpus and were *material* in almost half of the cases (n=876, 10.67%), followed by *relational* (n=495, 6.03%). In Table 5, we show the *process* occurrences in percentages (%) for each process category identified in the EOs expressed. Overall, all the processes were evenly spread in both positive and negative EO categories. *Material* (n=224, 43.2%) and *relational* (n=192, 37.0%) processes were the most recurrent for expressing EOs, often combined in the same EO category. Participants used *material* and *relational* processes similarly to express an *explicit negative EO* (n=101, 45.1%, and n=81, 42.2%, respectively), mostly for *explicit expression of negative appreciation* (eg “I was *working* [material] in the office last week and my diet *was* [relational] terrible”; n=69, 30.8%, and n=54, 28.1%, respectively) and e*xplicit or implicit expression of positive self-judgement* (eg, “On Friday I *did* [material] my Pilates classes and it *was* [relational] great after, as a miracle my back pain *disappeared* [material]”; n=101, 45.1%, and n=79, 41.2%, respectively). In both cases, participants introduced the situation with the material process and communicated their emotions about it with the relational process. In addition, *mental* and *behavioral* processes were used more often for *negative* (eg, “I *forgot* [mental] to take my multivitamin for 3 days last week” and “Things aren’t the same since before childbirth sometimes when I *sneeze* [behavioral]”; n=36, 62%, vs n=22, 28%, and n=16, 64%, vs n=9, 36%, respectively) than for *positive* EOs (eg, “I *feel* [mental] my sleep is getting better but I *think* [mental] that might be due to increasing my walking distance” and “…also *listening* [behavioral] to my body when I need rest and a cup of tea”). *Existential* (eg, “…however *there has been* [existential] a day or two were I didnt snack and that reflected in my energy levels and mood”) and verbal processes (eg “I have to *admit* [verbal] that our portion sizes would be much larger than these”) were marginally identified in 6 (1%) and 14 (3%) of all EOs in a similar proportion for negative and positive expressions (n=3, 50%, each and n=7, 50%, each, respectively). However, when the expressions were negative, participants only used these processes for the *explicit expression of negative appreciation* category.

**Table 3:** Participants’ demographics and program details.

Characteristic/detail		Participants
**Age (years), mean (SD)**		37.59 (3.69)
**BMI, mean (SD)**		25.82 (5.68)
**Program details, mean (SD)**		
	Days on program	80.76 (30.47)
	Coach sent messages	7.62 (1.82)
	User sent messages	2.27 (1.19)
**Goals of participants, n (%)**		
	Weight	7 (24.1)
	Physical activity	27 (93.1)
	Number of steps	21 (72.4)
	Diet	24 (82.8)
	Life	15 (51.7)

**Table 4:** Coding results expressed in number of occurrences, word count, and percentage of total word count.

Category		Occurrences, n (%)	Word count (N=8209), n (%)
**EOs^a^**		164 (100)	3478 (42.37)
	Implicit expression of negative feelings	12 (7.32)	351 (4.28)
	Implicit expression of negative appreciation (things, events, actions)	39 (23.78)	987 (12.02)
	Implicit expression of negative judgement (others or self)	4 (2.44)	104 (1.27)
	Pooled implicit negative EOs	55 (33.54)	1442 (17.57)
	Explicit expression of negative feelings	15 (9.15)	267 (3.25)
	Explicit expression of negative appreciation (things, events, actions)	20 (12.20)	317 (3.86)
	Explicit expression of negative judgement (others or self)	0	0
	Pooled explicit negative EOs	35 (21.34)	584 (7.11)
	Pooled negative EOs	90 (54.88)	2026 (24.68)
	Explicit or implicit expression of positive self-judgement	74 (45.12)	1481 (18.04)
**Message structure (n=148 messages)**			
	Opening	75 (34.40)	430 (5.24)
	Content	96 (44.04)	7077 (86.21)
	Closing	47 (21.56)	270 (3.29)
	Full structure (pooled opening, content, and closing)	38 (25.68)	4011 (48.86)
	Single (opening, content, or closing)	28 (18.92)	432 (5.26)
**Sentence structure (n=734 sentences)**			
	Declarative	697 (94.96)	7084 (86.30)
	Declarative, question function	4 (0.54)	76 (0.93)
	Imperative	14 (1.91)	88 (1.07)
	Interrogative	19 (2.59)	187 (2.28)
	Other	0	774 (9.43)
**Process**		1025 (100)	1839 (22.40)
	Behavioral	34 (3.32)	87 (1.06)
	Existential	10 (0.98)	24 (0.29)
	Material	430 (41.95)	876 (10.67)
	Mental	180 (17.56)	287 (3.50)
	Relational	325 (31.71)	495 (6.03)
	Verbal	46 (4.49)	85 (1.04)

^a^EO: empathy opportunity.

**Table 5:** Percentage (%) of occurrences per process category identified for each EO^a^ category.

Process (n=519 occurrences, 50.63%) found in the identified EOs (n=164)		Behavioral (n=25, 4.8%), n (%)	Existential (n=6, 1.2%), n (%)	Material (n=224, 43.2%), n (%)	Mental (n=58, 11.2%), n (%)	Relational (n= 192, 37.0%), n (%)	Verbal (n=14, 2.7%), n (%)
**EOs (n=164 occurrences)**							
	Explicit expression of negative appreciation (things, events, actions)	3 (12.0)	3 (50.0)	69 (30.8)	13 (22.4)	54 (28.1)	6 (43.0)
	Explicit expression of negative judgement (others or self)	2 (8)	0	7 (3.1)	2 (3.4)	8 (4.2)	1 (7.0)
	Explicit expressions of negative feelings	4 (16.0)	0	25 (11.2)	7 (12.1)	19 (9.9)	0
	*Pooled explicit negative EOs*	9 (36.0)	3 (50.0)	101 (45.1)	21 (36.2)	81 (42.2)	7 (50.0)
	Implicit expression of negative appreciation (things, events, actions)	5 (20.0)	0	16 (7.1)	3 (5.2)	17 (8.9)	0
	Implicit expression of negative judgement (others or self)	0	0	0	0	0	0
	Implicit expressions of negative feelings	2 (8.0)	0	7 (3.1)	11 (19.0)	15 (7.8)	0
	*Pooled implicit negative EOs*	7 (28.0)	0	22 (9.8)	15 (25.9)	33 (17.2)	0
	*Pooled negative EOs*	16 (64.0)	3 (50.0)	123 (54.9)	36 (62.1)	113 (58.9)	7 (50.0)
	Explicit or implicit expression of positive self-judgement	9 (36.0)	3 (50.0)	101 (45.1)	22 (37.9)	79 (41.1)	7 (50.0)

^a^EO: empathy opportunity.

### Transitivity Analysis

When we performed a transitivity analysis, the participant roles varied according to the process type. As shown in Table 4, *material* processes dominated the text corpus (n=876, 10.67%), followed by *relational* (n=495, 6.03%). *Relational* processes are used for either characterizing, including a carrier and an attribute as components of the system, or identifying, involving a value and a token. *Material* processes, on the other hand, include an actor (participant), and some demand a goal, while others do not [54]. In addition to these grammatical roles, we categorized findings from this analysis thematically to supplement the meanings expressed. We present the results from transitivity and thematic analyses in Tables 6-11. Most (n=300, 92.2%) *relational* processes were *attributive* (eg, “Your links *were* very helpful”). The remaining 7.8% (n=25) were *identifying* (“My starting weight was 51.5kg”). The most frequent themes were food and diet (n=63, 19.3%), well-being (n=60, 18.2%), and physical activity (n=44, 13.5%). Similarly, *material* processes frequently (n=217, 70.4%) had the user as the *actor*, and although their goals were widely spread, the most common categories were food and diet (n=196, 54.9%), physical activity (n=96, 26.9%), and goals (n=40, 11.2%). For example, “I *open* the dates *put* a bit of peanut butter in them, then *put* them in the freezer to harden” and “I've *added* a pelvic floor exercise goal.” In contrast, *mental* processes (n=287, 3.5%) involve a *senser* and a *phenomenon* in the transitivity system. This corpus showed a predominance (n=84, 93.3%) of the user as the *senser* and food and diet (n=24, 26.1%), well-being (n=16, 18.9%) and physical activity (n=13, 14.8%) as the *phenomenon* (eg, “I have *included* new snacks like olives” and “I *decided* to have a go with cross trainer”). In *verbal* clauses, a *sayer* directs a message to a *receiver*. In this corpus, despite its low occurrence (n=85, 1.0%), the most frequent *sayer* was the user (n=19, 55.9%) and the *receiver* was usually a health care professional (HCP; n=5, 38.5%; eg, “I *talked* with my GP about the pains”). The most common thematic, as in the other processes, was food and diet (n=9, 20.5%), with an identical occurrence to well-being (n=9, 20.5%).

**Table 6:** Transitivity analysis results for material processes.

Processes, grammatical roles, and themes		Occurrences, n (%)
**Actor (n=308)**		
	User	217 (70.4)
	Not human	63 (20.7)
	We	11 (3.6)
	Another person	6 (1.8)
	Coach	5 (1.6)
	User’s HCP^a^	4 (1.3)
	User’s partner	2 (0.6)
**Goal (n=357)**		
	Food and diet	196 (54.9)
	Physical activity	96 (26.9)
	Goals	40 (11.2)
	Other (something, nothing, anything, things)	17 (4.6)
	Other (place, object, pain, work, mood, medicine, body part)	5 (1.4)
	Message (user or coach sent)	2 (0.5)
	Person (user, coach, baby, HCP)	1 (0.2)
	App	1 (0.2)

^a^HCP: health care professional.

**Table 7:** Transitivity analysis results for relational processes.

Processes, grammatical roles, and themes		Occurrences, n (%)
**Attributive**		300 (92.2)
	With a carrier	266 (81.7)
	Without a carrier	34 (10.4)
Identifying		25 (7.8)
**Themes (n=325)**		
	Food and diet	63 (19.3)
	Well-being	60 (18.2)
	Physical activity	44 (13.5)
	Goals	30 (9.1)
	Pregnancy and baby	27 (8.1)
	Pain	25 (7.8)
	Stress	21 (6.4)
	Work	16 (4.7)
	App	14 (4.4)
	Coach messages	9 (2.7)
	Mood and emotions	8 (2.4)
	User messages	5 (1.4)
	Weather	3 (1.0)
	App	1 (0.2)

**Table 8:** Transitivity analysis results for mental processes.

Processes, grammatical roles, and themes		Occurrences, n (%)
**Senser (n=90)**		
	User	84 (93.3)
	Coach	3 (3.2)
	Doctor	2 (2.1)
	User’s partner	1 (1.1)
**Phenomenon (n=90)**		
	Food and diet	24 (26.1)
	Well-being	16 (18.2)
	Physical activity	13 (14.8)
	Goals	9 (10.2)
	App	9 (10.2)
	Pain	9 (10.2)
	Planning	4 (4.6)
	Baby	3 (3.4)
	Coach messages	2 (2.3)

**Table 9:** Transitivity analysis results for behavioral processes.

Processes, grammatical roles, and themes		Occurrences, n (%)
**Behaver (n=15)**		
	User	13 (94.3)
	You	1 (2.9)
	We	1 (2.9)
**Themes (n=35)**		
	Food and diet	13 (38.2)
	Physical activity	5 (14.7)
	Pain	3 (8.8)
	App	3 (8.8)
	Goals	3 (8.8)
	Pregnancy and baby	3 (8.8)
	Well-being	3 (8.8)
	Coach messages	1 (2.9)

**Table 10:** Transitivity analysis results for verbal processes.

Processes, grammatical roles, and themes		Occurrences, n (%)
**Sayer (n=34)**		
	User	19 (55.9)
	Coach	12 (35.3)
	HCP^a^	2 (5.9)
	We	1 (2.9)
**Receiver (n=13)**		
	HCP	5 (38.5)
	User	4 (30.8)
	Coach	4 (30.8)
**Themes (n=46)**		
	Food and diet	9 (20.5)
	Well-being	9 (20.5)
	Coach messages	7 (15.4)
	Pain	6 (12.8)
	Physical activity	6 (12.8)
	Goals	4 (7.7)
	User messages	2 (5.1)
	App	1 (2.6)
	Pregnancy and baby	1 (2.6)

^a^HCP: health care professional.

**Table 11:** Transitivity analysis results for existential processes.

Processes, grammatical roles, and themes		Occurrences, n (%)
**Themes (n=10)**		
	Physical activity	3 (30.0)
	Food and diet	3 (30.0)
	Stress	2 (20.0)
	App	1 (10.0)
	Pregnancy and baby	1 (10.0)

## Discussion

### Principal Findings

Empathy and SFL approaches, as we hypothesized in the introduction, can be successfully combined. Our findings show that the SFL categories we explored in the transitivity analysis correspond to and supplement Pounds' EOs. Our findings reveal interesting meanings that originate from the user's linguistic choices whenever they express an EO, particularly when these are “hidden” in an implicit form. Since HCPs frequently overlook these in patient-provider communication, identifying and responding to them optimally is critical for successful health interventions. To the best of our knowledge, no other researchers have previously presented this novel perspective.

Overall, our results show that the users expressed negative EOs more often than positive ones (74 vs 90), of which 60% (55 of 90) were expressed implicitly. Given the context of our data set—a coaching program in which pregnant women communicate with a coach who supports them throughout their journey—the existence of negative EOs is not surprising. We frequently use negative statements to draw attention to a problem that we expect the receiver to empathize with or assist us with. Positive EOs, on the other hand, are less common because they do not serve the purpose of seeking support. However, they do provide a chance for the coach to praise the user’s behavior [53]. Moreover, the user’s preference for implicit EOs could be due to a polite relationship with their coaches, which would prevent them from making too negative statements. The absence of *explicit expressions of negative judgment about others or self* could support this interpretation. Such insights could be useful for coaches to detect empathic expressions and support users further. Moreover, our results from the message and sentence structure analyses indicated that most of the wording used was found in the content section of the message with a predominant use of a declarative structure. We explored a relationship between the empathy categories and the message structure, and between the empathy categories and the sentence structure. Such analyses showed no variability in the data across categories; hence, we chose not to include them in this paper. Nevertheless, the predominance of this sentence structure finding is expected, as the use of statements prevails in lengthier and more monological, narrational stretches of communication, which most of these messages were. The scarce presence of interrogative sentences (2.28%) shows that these users were not posing questions and asking for help. Nonetheless, a more qualitative analysis of interrogative occurrences could provide a deeper understanding of these linguistic choices. We added a fourth category during our analysis to account for those sentences where a question (interrogative function) was atypically realized through a statement (declarative structure), representing 0.93% of the sentences in the corpus. A functional interpretation of this phenomenon could be that users were moderating their queries to be less imposing and less direct. The short interaction time (3 months) can also explain this declarative choice, indicating an insufficient time allowed to develop a coach-user relationship. At the start of the program, users met their coach during a synchronous call followed by a small number of purely asynchronous interactions (the mean for coach-sent messages was 7.62 and user-sent messages was 2.27). Participants could be afraid or not feel the confidence to actively ask for information or help due to an insufficient or too polite relationship with their coach. Another reason could be low trust on or missing information about the coach’s ability to support them.

Our transitivity analysis showed that the users were the main participants in the clause (eg, material processes described their lifestyle actions, such as food and diet, physical activity, and goals), such as cooking, eating, or exercising, as this was a lifestyle, goal setting–based coaching program. Additionally, these results were in line with the characteristics of the EOs detected: material processes were predominately used for *expressions of positive self-judgment* (eg, “I am *eating* healthy and *doing* long walks every day”) or *implicit expressions of negative appreciation* (eg, “I only *had* one Panadol for pain management, but it *does not work* that well”); mental processes disclosed negative feelings (eg, “I *feel* extremely tired and struggling to get 10 thousand step per day”), and negative appreciations were realized through attributive, relational processes (eg, “my snacking *has been* desperate”). These are highly interesting findings for health behavior change programs that have the potential to contribute to promoting user outcomes [75]. The information shared by the user, being explicit or implicit, helps the coach understand the user’s perspectives and coaching needs. Although explicit expressions are easy to detect, a more efficient detection and understanding of implicit expressions will contribute to better coaching support. Our insights will provide guidance on the most empathic coach responses and serve to determine the optimal text message–based coach responses in telehealth interventions. Because of its relationship to the EO categories, transitivity analysis opens a range of opportunities for improving patient care overall. Our perspectives for these findings include further exploration of the empathy and the linguistic elements (SFL) found in the coaches’ optimal responses and their connection with user outcomes. Moreover, an association with the BCTs used by the coaches in response to these messages could provide additional insights to boost the impact of digital lifestyle text-based interventions.

### Comparison With Prior Work

As we previously described in the Introduction section of this paper, prior work has mainly studied empathy and linguistics in text-based communication separately. Empathy is an important element in the patient-provider relationship that improves patient outcomes [47]. Some authors have measured empathy in a digital setting with surveys [76], with different indexes or scales [77], or as a predefined element in broader coding systems [78]. Other research work has focused on a computational approach to automatize empathy detection (eg, in digital mental health services [79,80]). With regard to linguistics, there has been an increasing interest in digital communication [81], and researchers have applied different linguistic perspectives, such as digital conversation analysis (CA) [82] and SFL. Both CA and SFL perspectives have been applied to digital contexts, such as social media and digital consultations [83]. However, Pounds [53] was the first one to recently define and demonstrate the use of empathy appraisal categories in text-based, patient-provider interactions. The appraisal framework generally studies the meaning negotiation among the speakers, using every utterance to align or misalign with others. In SFL, this framework describes the linguistic resources that the speakers use to construe their social experience and build an intersubjectivity with the recipient, contributing to the interpersonal metafunction [84]. Furthermore, according to Martin and White [85], the appraisal system is organized in 3 domains used to negotiate and modulate emotions, judgements, and valuations: *engagement*, *attitude*, and *graduation*. The *attitude* is the system of meanings represented by the feelings expressed. *Graduation* intensifies or diminishes this representation of meanings. *Engagement* reflects the commitment of the speaker to the appraisal expressed. These appraisal system domains are further explained by Martin [86] as an expansion of the theoretical and descriptive focus of SFL described by Halliday and serve to analyze the speakers’ feelings. Pounds’ empathy appraisal categories are grounded on this research work and were later suggested for their combination with SFL categories by Pounds herself and De-Pablos Ortega [42]. We contacted Pounds and De-Pablos Ortega for research collaboration. However, they confirmed that they had discontinued their work on the topic. Thereby, we are the first to code a text-based health interaction using both an empathy and an SFL approach. This pilot study served to assess whether these 2 methodologies were compatible, with promising results. We will include further features previously used in text-message coding, such as sentiment analysis, in our future research. Some software can perform automatic sentiment analysis (eg, through word rating [87]), while other authors have resorted to more elaborate machine learning and algorithms for more accurate results [88,89]. Furthermore, coding for additional elements (eg, emojis or modality) could inform the negotiation of interpersonal roles, such as the user-coach relationship, and be paired with the EO displayed [73].

### Limitations

Given the small sample size (n=148), our results should be carefully observed. We aimed to test the combination of empathy and linguistic approaches for text messages analysis. Our findings are preliminary and part of a broader project that will continue exploring methodological possibilities in asynchronous communication analysis.

### Conclusion

Our transitivity analysis supports the combination of an empathy and a linguistic (SFL) approach. The processes and their related elements correlate with the empathy categories identified in the corpus. These are promising results for future coding in asynchronous, online interactions. Our study findings shed light on the empathy and linguistic characteristics present in text message–based coaching. We draw attention to the meanings of patient EOs, such as implicitly seeking help or praise, because research shows that HCPs frequently miss these opportunities. Their identification and management have significant implications for the coach-user relationship and to improve coach training in the future. Our next steps will be to study the coaches’ messages and to explore the coach-user relationship-building process. We will code the coaches’ messages for linguistic choices (SFL) and how they respond to the EOs presented by the users. Additionally, we will link our results with user outcomes in this lifestyle coaching program during pregnancy when at risk of GDM. This future research will allow for the formulation of optimal coach empathic responses.

## Abbreviations

AI: artificial Intelligence
BCT: behavior change technique
CA: conversation analysis
EO: empathy opportunity
GDM: gestational diabetes mellitus
HCP: health care professional
IHC: integrative health coaching
NCD: noncommunicable disease
PCC: patient-centered communication
SFL: systemic functional linguistics

## Data Availability

The data sets generated and analyzed during this study are not publicly available due to the presence of sensitive information, such as names and places, in the user messages analyzed. Researchers who wish to access the full data set may be granted access upon reasonable request to the corresponding author.
